# Neuro-Behçet’s Disease in a Middle Eastern Male: A Rare and Challenging Diagnosis

**DOI:** 10.7759/cureus.85628

**Published:** 2025-06-09

**Authors:** Madeena Mahmood, Saqib Iqbal, Abbas Badawi

**Affiliations:** 1 Hospital Medicine, Mersey West Lancashire Teaching Hospitals NHS Trust, Liverpool, GBR; 2 Respiratory Medicine, Mersey West Lancashire Teaching Hospitals NHS Trust, Liverpool, GBR

**Keywords:** behçet's disease, diagnosis of rare cases, genital ulcers, hla-b51, mouth ulcers, rheumatology, vascular neurology, vasculitis

## Abstract

Neuro-Behçet’s Disease (NBD) is a rare neurological complication of a chronic, multisystem inflammatory condition. It typically manifests several years after the onset of initial non-neurological symptoms, such as oral and genital ulcers, uveitis, and dermatological or gastrointestinal issues. Neurological involvement occurs infrequently and can be challenging to diagnose in its early stages.

This case describes a young adult who presented with progressive neurological symptoms, following a long-standing history of systemic inflammatory features. The diagnostic process included neuroimaging that revealed signs consistent with intracranial abnormalities and vascular involvement. Following clinical and radiological assessment, a diagnosis of NBD was made, and appropriate treatment was initiated.

This case underscores the importance of considering a broad differential diagnosis when evaluating patients with complex symptom profiles, and the value of a comprehensive, patient-centred approach to avoid unnecessary investigations and reduce the length of hospital stay.

## Introduction

Behçet's Disease (BD) is an uncommon, long-lasting inflammatory condition that affects a variety of systems in the body. Some key symptoms consist of repeated oral and genital sores, uveitis, and skin lesions, which may affect vessels of various sizes [1]. BD is predominantly found in the “Silk Road” area - the Middle East, Asia, and the Mediterranean - especially in Turkey, where it has the highest prevalence. The illness is closely linked to carriers of HLA-B51/B5, who face a heightened risk of developing BD [2].

Unlike other autoimmune diseases, BD does not have specific autoantibodies, with cell-mediated immunity being crucial to its pathogenesis [3]. Factors contributing include circulating immune complexes, antibodies against endothelial cells, and endothelial dysfunction [3]. The illness tends to be more intense in males and younger people, with the majority of cases arising sporadically [4].

Neuro-Behçet's Disease (NBD) develops in less than 10% of instances, usually appearing approximately five years after the onset of initial symptoms. In 80% of NBD cases, the brainstem is affected, resulting in cerebellar, pyramidal, and sensory impairments. Cerebrospinal fluid (CSF) is usually free from any contaminants, although protein or cell levels may be increased [5].

This case study examines an 18-year-old Syrian male who presented with persistent headaches, notable unexplained weight loss, and blurred vision. Even though these symptoms are nonspecific, additional examinations resulted in a diagnosis of NBD. Our objective is to underscore the diagnostic difficulties of BD, examine differential diagnoses, and stress the significance of a collaborative approach for addressing atypical presentations.

## Case presentation

A 17-year-old male presented to the Emergency Department (ED) with a two-week history of blurred vision, a five-month history of daily headaches, a 20 kg weight loss over the last couple of months, and mouth ulcers for the last five years. Observations and blood tests (full blood count and liver function tests) were satisfactory, apart from a C-reactive protein (CRP) level of 31 mg/L (normal value: 0-9 mg/L). A magnetic resonance imaging (MRI) head scan was performed, which identified bilateral papilledema. It was assumed that, due to the patient’s history of being a Syrian immigrant and his presentation, he may have tuberculosis (TB). A lumbar puncture was performed, which revealed a CSF protein level of 0.62 g/L (normal range: 0.15-0.45), which was otherwise satisfactory. Further tests for *Treponema*, human immunodeficiency virus (HIV), hepatitis B virus (HBV), anti-neutrophil cytoplasmic antibodies (ANCAs), hepatitis, and TB screening were sent, which have all returned negative to date. 

**Figure 1:**
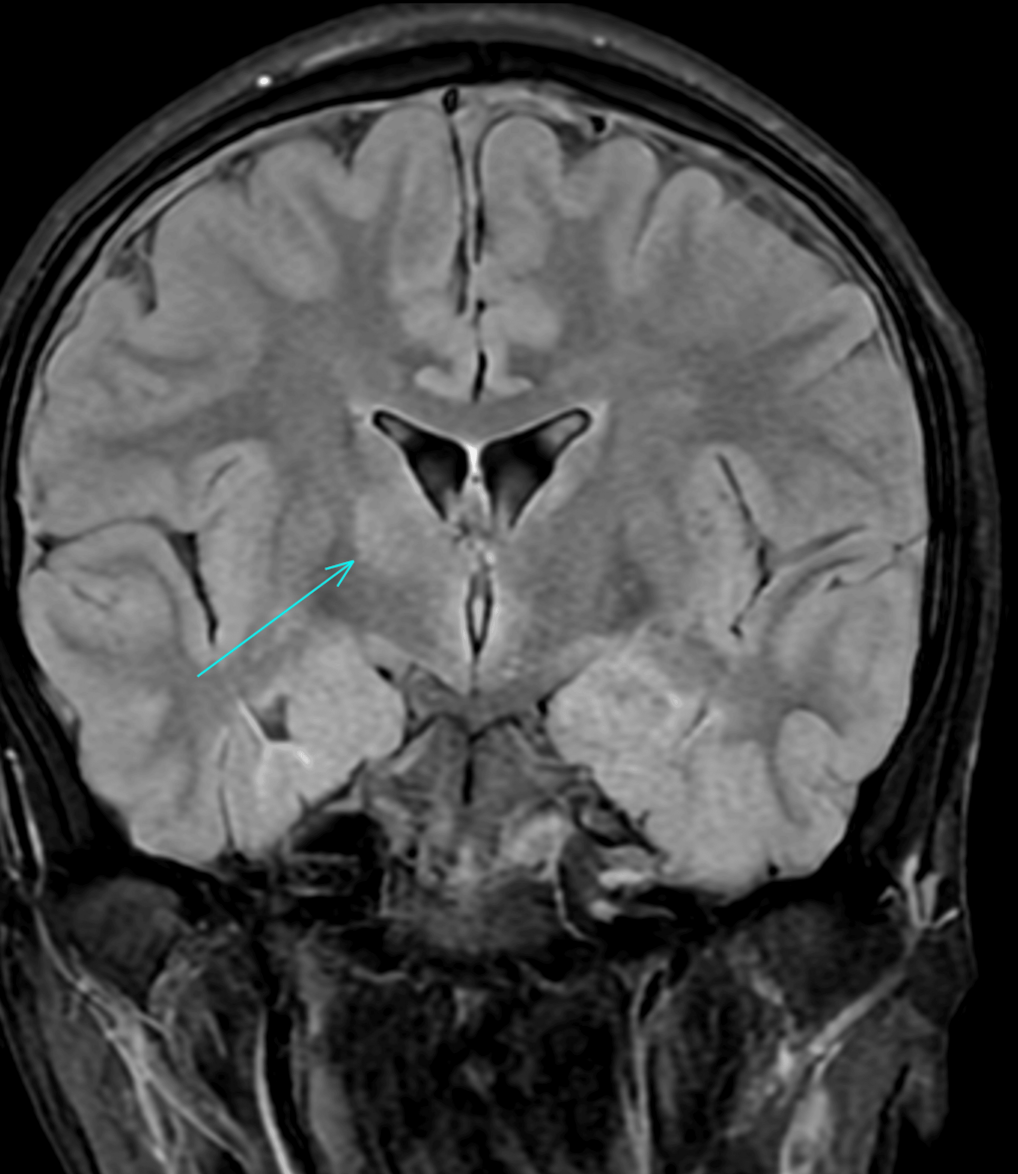
MRI head scan highlighting inflammatory changes (arrow, coronal view) MRI, magnetic resonance imaging

**Figure 2:**
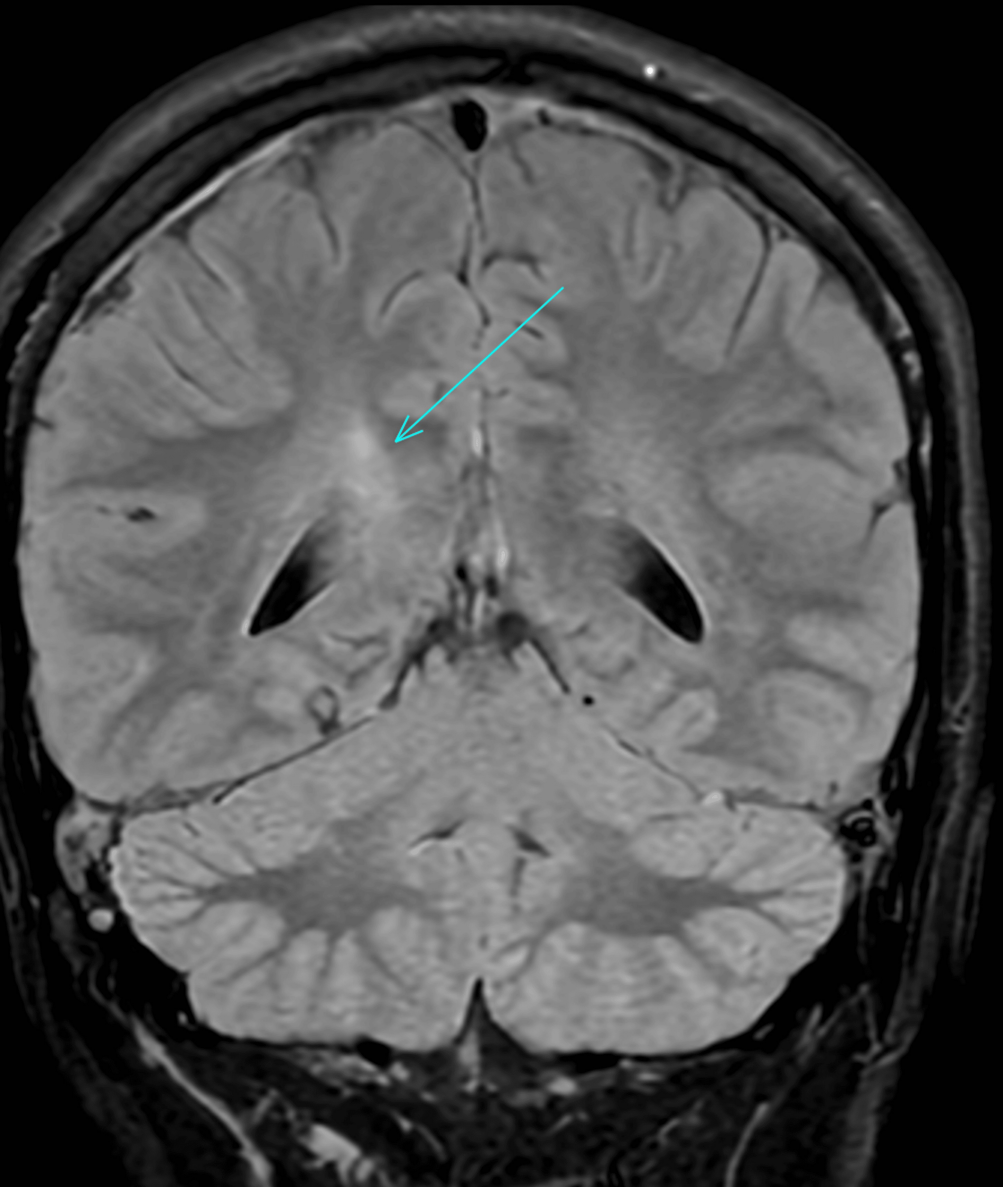
MRI head scan highlighting inflammatory changes (arrow, axial view) MRI, magnetic resonance imaging

**Figure 3:**
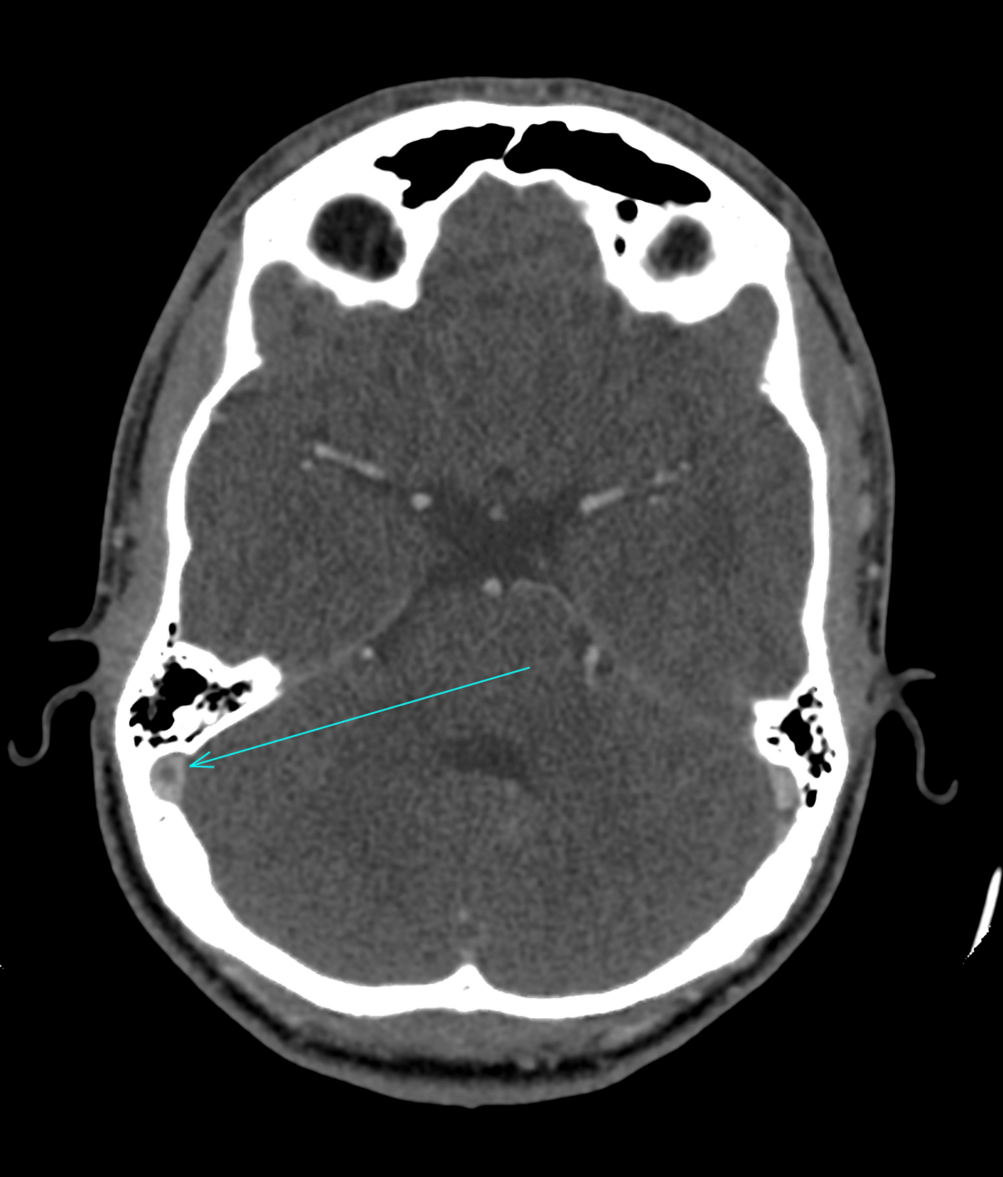
CT venogram scan highlighting thrombosis (arrow, axial view) CT, computerised tomography

**Figure 4:**
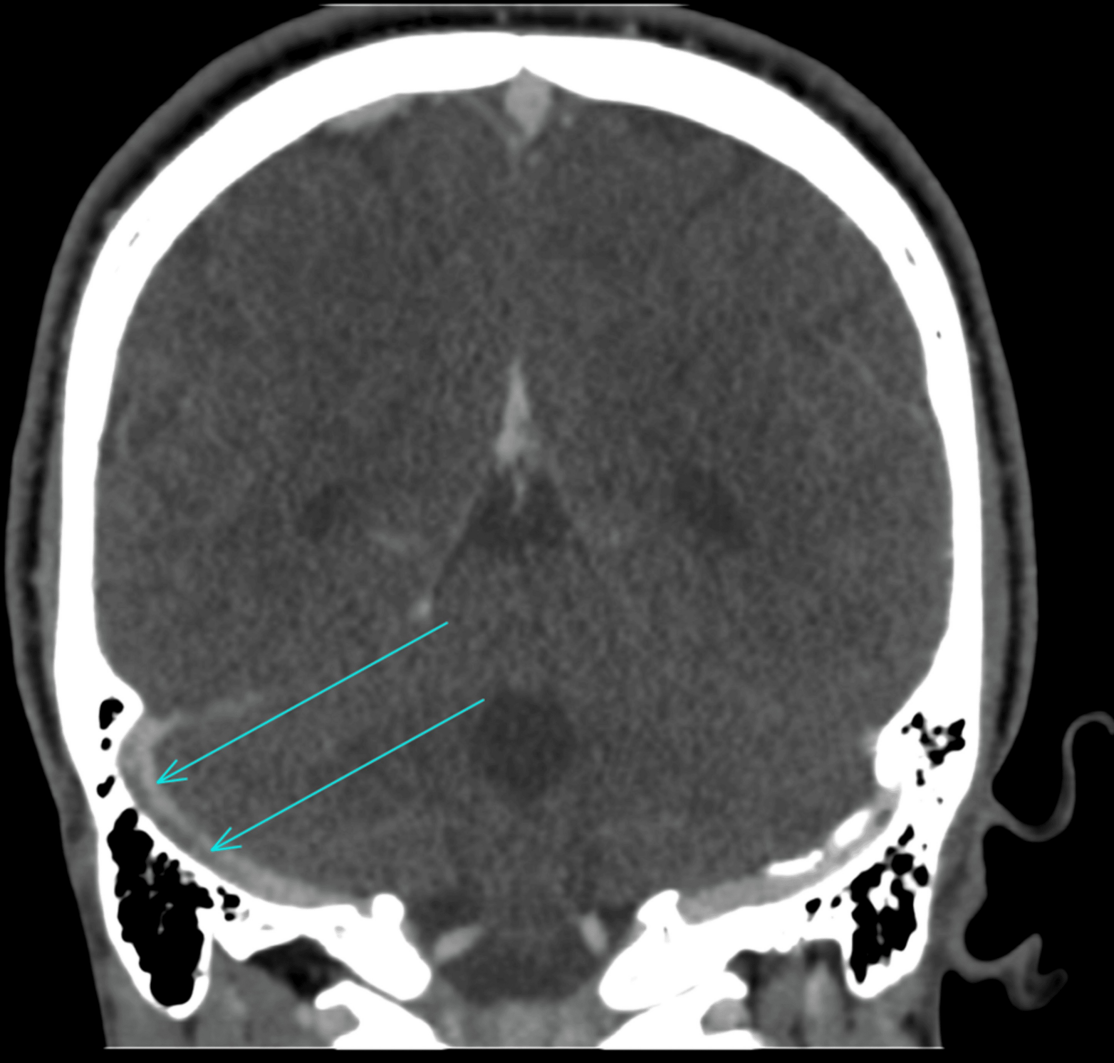
CT venogram scan highlighting thrombosis (arrows, coronal view) CT, computerised tomography

A computerised tomography (CT) scan of the neck, thorax, abdomen, and pelvis was performed, which showed evidence of a slightly prominent, enhancing left supraclavicular node. An ultrasound of the neck indicated that the lymph nodes were within normal limits; therefore, a fine needle aspiration was not performed. An MRI of the head was performed, which raised the possibility of NBD due to a dural venous sinus thrombus that was identified. The MRI indicated a high T2/FLAIR (fluid-attenuated inversion recovery) signal along the right corticospinal tract - including the internal capsule, cerebral peduncle, upper pons, thalamus, and splenium - with milder involvement on the left. Multiple deep and periventricular white matter lesions, exceeding age-related expectations, were suggestive of an inflammatory process (Figures 1-2). Due to these MRI findings, a CT venogram was performed, which highlighted thrombi in the anterior superior sagittal sinus, left transverse sinus, right transverse/sigmoid sinus, and the right jugular bulb/internal jugular vein (IJV) (Figures 3-4). He was seen by neurology and rheumatology while an inpatient. A diagnosis of NBD was ultimately established based on clinical assessment by the rheumatology team. It was also discovered, after taking a new history during his hospital stay, that a close relative had previously been diagnosed with BD, providing additional support for the diagnosis. 

The patient was started on dabigatran for the thrombi and, eventually, methylprednisolone intravenously, which was then transitioned to oral prednisolone after being reviewed by the rheumatology team. The prednisolone was initially prescribed at 50 mg for two weeks, then reduced by 10 mg every two weeks until 30 mg, and finally reduced by 5 mg every two weeks until a maintenance dose of 20 mg was achieved. He was also given betamethasone soluble tablets to help with his mouth ulcers.

After the diagnosis of NBD was established, he was discharged following an inpatient stay of around three weeks. He was discharged with outpatient neurology and rheumatology follow-up appointments to manage his condition.

## Discussion

BD is an autoimmune vasculitis that typically presents with a combination of recurrent oral ulcers, genital ulcers, uveitis, and cutaneous lesions. Given the clinical presentation, the patient's sexual history was considered upon admission, and investigations for hepatitis and HIV were conducted - both of which returned negative results. Suspicion of TB also arose due to the patient’s social and medical history. 

Diagnosing NBD can be challenging, as its clinical presentation may overlap with other conditions that are included in the differential diagnosis, such as multiple sclerosis, thromboembolic stroke, meningoencephalitis, and reversible cerebral vasoconstriction syndrome. The involvement of the central nervous system (CNS) falls into parenchymal and non-parenchymal subtypes. The parenchymal subtype is common, with symptoms typically resulting from the involvement of the spine and brainstem. The non-parenchymal type includes conditions such as cerebral venous sinus thrombosis (CVST) and arterial involvement [6].

Neurological symptoms in patients with BD occur in less than 10% of cases and are known to be more frequent among males [7]. NBD can result in a wide range of CNS deficits and is primarily linked with vascular thrombosis affecting the brainstem, basal ganglia, or spinal cord [8]. While symptoms of NBD can vary and are often related to focal parenchymal lesions, the primary indications for obtaining an MRI in this case were the patient's severe headaches, blurred vision, and unexplained rapid weight loss.

Similarly to the findings in our case, BD has been identified as a contributing factor in the development of CVST. Although the initial MRI indicated evidence of CVST, the patient had not yet been diagnosed with BD at that point in time. It wasn't until the CT venography scan results were reviewed, together with a more thorough history - including the identification of a family member with BD - alongside the integration of the patient's systemic and neurological symptoms, that a diagnosis of NBD came into focus. 

The patient was started on a high-dose corticosteroid regimen in combination with an anticoagulant to achieve remission. Shortly after beginning steroid therapy, the patient experienced noticeable clinical improvement and a return of appetite.

He was discharged with a follow-up with the rheumatologist and neurologist team to manage his condition locally as an outpatient, where an immunomodulator could potentially be introduced under the guidance of the rheumatology team. Biologics such as infliximab and adalimumab are engineered to inhibit the activity of tumour necrosis factor-alpha (TNF-α), a key driver of inflammation. By blocking TNF-α, these therapies help reduce inflammation and alleviate the symptoms associated with NBD.

## Conclusions

NBD can be difficult to recognise due to its varied presentation and overlap with other neurological conditions. NBD can resemble other central system vasculitides or demyelinating diseases, which subsequently results in diagnostic delay. When individuals show unexplained neurological symptoms along with specific brain lesions and vascular changes, NBD should be part of the differential diagnosis. In such cases, examining the patient for broader systemic signs - such as recurring mouth or genital sores, eye inflammation, and skin issues - can be central. Identifying these features early can help guide clinicians toward an accurate diagnosis and a more effective treatment approach.
